# Evaluation of Treatment Effects of en Masse Mandibular Arch Distalization Using Skeletal Temporary Anchorage Devices: A Systematic Review

**DOI:** 10.7759/cureus.71171

**Published:** 2024-10-09

**Authors:** Nisshitha R Setvaji, Shantha Sundari

**Affiliations:** 1 Orthodontics and Dentofacial Orthopaedics, Saveetha Dental College and Hospital, Saveetha Institute of Medical and Technical Sciences, Saveetha University, Chennai, IND

**Keywords:** class iii malocclusion, en-masse mandibular arch distalization, en-masse retraction, orthodontic anchorage, orthodontic bone screws

## Abstract

Mandibular arch distalization using temporary anchorage devices (TADs) is effective in correcting borderline Class lll cases without surgery. This review analysed the existing literature evaluating the dental, skeletal, and soft tissue changes after en masse mandibular arch distalization using TADs. We followed PRISMA guidelines and registered this review in PROSPERO database CRD42023450524. PubMed, Google Scholar, Cochrane, Web of Science, and SCOPUS databases were searched. Orthodontic patients requiring en masse mandibular distalization using TADs were compared with different modalities of distalization. Dental changes were evaluated along with skeletal and soft tissue changes and treatment duration. Randomized controlled trials (RCTs), cohort studies, longitudinal studies, and retrospective studies were eligible to be included. The risk of bias was assessed using ROBINS-I tool ("Risk Of Bias In Non-randomized Studies-of Interventions"). Of 1764 identified records, seven studies (one non-RCT, one prospective clinical trial, and five retrospective cohort studies) were included. One study had a low risk of bias, three had moderate bias, two had serious bias, and one had critical bias. All studies showed molar distalization and distal tipping with significant retraction of the lower lip. Miniplates showed greater distalization compared to miniscrews. Interradicular miniscrews exhibited the least distal tipping. Changes in SNB, ANB, occlusal plane angle, and mandibular plane angle were reported. Retromolar screws and ramal plates reported reduced treatment duration. TADs are effective in en masse distalization of mandibular dentition with intrusive or extrusive effects.

## Introduction and background

Common treatment strategies for Class III malocclusion in adult patients include extractions, surgical interventions, or camouflage by upper incisor proclination and lower incisor retroclination [1]. Several approaches towards treating moderate class III patients include multiloop edgewise archwire with intermaxillary elastics therapy, lip bumpers, modified fixed reverse twin block appliances, high-pull J-hook headgear, distal extension lingual arch, and others [2-5]. These techniques provide a comparatively stable occlusion with adequate overjet and overbite. Nevertheless, it was inevitable to experience unfavourable side effects such as upper incisor proclination, upper molar extrusion, and distal tipping of the lower molars. 

Utilizing skeletal anchorage devices for distalization of the mandibular dentition has recently proven to be an effective treatment plan for the correction of borderline Class lll cases, especially with the non-extraction treatment protocol gaining popularity [6-8]. Temporary anchorage devices (TADs) produce mandibular distalization without any of the undesirable side effects [9,10]. Alternatively, for Class lll patients who are disinclined towards surgery, distalization of the mandibular dentition is a viable option [11]. 

TADs, including miniscrews, miniplates, bone screws, and ramal implants, are not dependent on patient compliance and are widely accepted by adult patients [12]. For mandibular distalization, TADs are placed in the buccal shelf areas, retromolar areas, interradicular areas, and ramus [13-15]. Previous case reports illustrate the efficiency of TADs in distalization of the whole dentition. Sugawara et al. used titanium anchor plates at the anterior border of the ramus to distalize the lower dentition [13]. Chung et al. used mini implants to correct Class lll malocclusion with a midline deviation [15]. Nevertheless, there are not many statistical clinical studies that compare the therapeutic outcomes of TADs positioned at various placement sites. Hence, the objective of this systematic review is to summarize the current evidence on dental, skeletal, and soft tissue changes following en masse mandibular arch distalization using TADs placed at various sites.

## Review

This review adhered to the Preferred Reporting Items for Systematic Reviews and Meta-Analyses (PRISMA) guidelines in conducting the systematic analysis. The main research question was defined in PICO format (Table 1).

**Table 1:** PICO format. TADs: temporary anchorage devices; PICO: population, intervention, comparison, and outcomes; RCTs: randomized controlled trials.

Structure	Definition
Population	Orthodontic patients requiring en masse mandibular arch distalization
Intervention	Skeletal TADs
Comparison	Different modalities of mandibular arch distalization and different locations of TAD placement
Outcomes	Primary: Mandibular molar and incisor distalization and vertical movement Secondary: Skeletal and soft tissue changes, treatment duration
Study Design	RCTs, cohort studies, longitudinal studies, and retrospective studies were eligible to be included

The review protocol was registered in the PROSPERO database under CRD42023450524. The methodology was established in advance of the review, adhering to the recommendations stated in the Cochrane Handbook. The selection criteria that were utilized to include the articles in the review are displayed in Table 2.

**Table 2:** Eligibility criteria used for the study selection. TADs: temporary anchorage devices; RCTs: randomized control trials.

Category	Inclusion criteria	Exclusion criteria
Population	Patients undergoing fixed orthodontic treatment requiring total mandibular arch distalization	Patients undergoing only anterior teeth retraction. Patients with systemic pathologies or pre-existing periodontal diseases, syndromic patients.
Intervention	Skeletal TADs such as extra-alveolar miniscrews, interradicular miniscrews, miniplates, ramal plates, etc.	Other methods of mandibular arch distalization without the use of TADs
Comparison	Different modalities of mandibular arch distalization and different locations of TAD placement	
Outcome	Primary outcomes: dental changes; secondary outcomes: skeletal and soft tissue changes and treatment duration	
Study design	RCTs, non-RCTs, cohort studies	Abstracts, animal studies, case studies, remarks and editorial letters, narrative reviews, systematic reviews, in vitro studies, and studies published in languages other than English

Search strategy

MEDLINE (through PubMed), the Cochrane Library, SCOPUS, Google Scholar, and Web of Science were among the electronic databases that were searched until May 2023. Detailed search strategies were developed based on the search syntax of each database (Table 3).

**Table 3:** Search strategy applied for study identification.

Database	Search query	Total count
PubMed	(((((((total arch) OR (whole arch)) OR (distalisation)) OR (distalization)) OR (distal movement)) OR (en masse)) OR (mandibular arch)) AND (((((((((((((((((((((((((temporary anchorage devices)) OR (TADs)) OR (mini screws)) OR (mini plates)) OR (mini implants)) OR (buccal shelf screws)) OR (buccal shelf implants)) OR (ramal implants)) OR (ramal screws)) OR (ramal plates) ) OR (retromolar implants)) OR (retromolar screws)) OR (retromolar plates)) OR (interradicular implants)) OR (interradicular screws)) OR (intra alveolar implants)) OR (intra alveolar screws)) OR (extra alveolar screws)) OR (extra alveolar implants)) OR (orthodontic screws)) OR (orthodontic implants)) OR (orthodontic mini implants)) OR (orthodontic mini screws))) AND (((((treatment effects)) OR (treatment outcomes)) OR (treatment changes)) OR (treatment duration))	669
Google Scholar	Mandibular arch AND en masse AND distalization AND temporary anchorage devices AND treatment effects OR miniscrews OR mini implants OR miniplates OR duration	833
Scopus	Mandibular AND arch AND distalization AND retraction AND miniscrews AND temporary AND anchorage AND devices AND treatment	109
Cochrane Library	Mandibular arch AND distalization OR temporary anchorage devices AND treatment	135
Web of Science	Mandibular arch AND distalization AND miniscrews AND treatment	18

All unpublished theses, case reports, and incomplete trials were excluded. No other language studies were identified. The bibliography of each qualified article was examined for further studies. Eligibility was assessed by both authors (N.R.S. and S.S.).

Study selection

**Figure 1:**
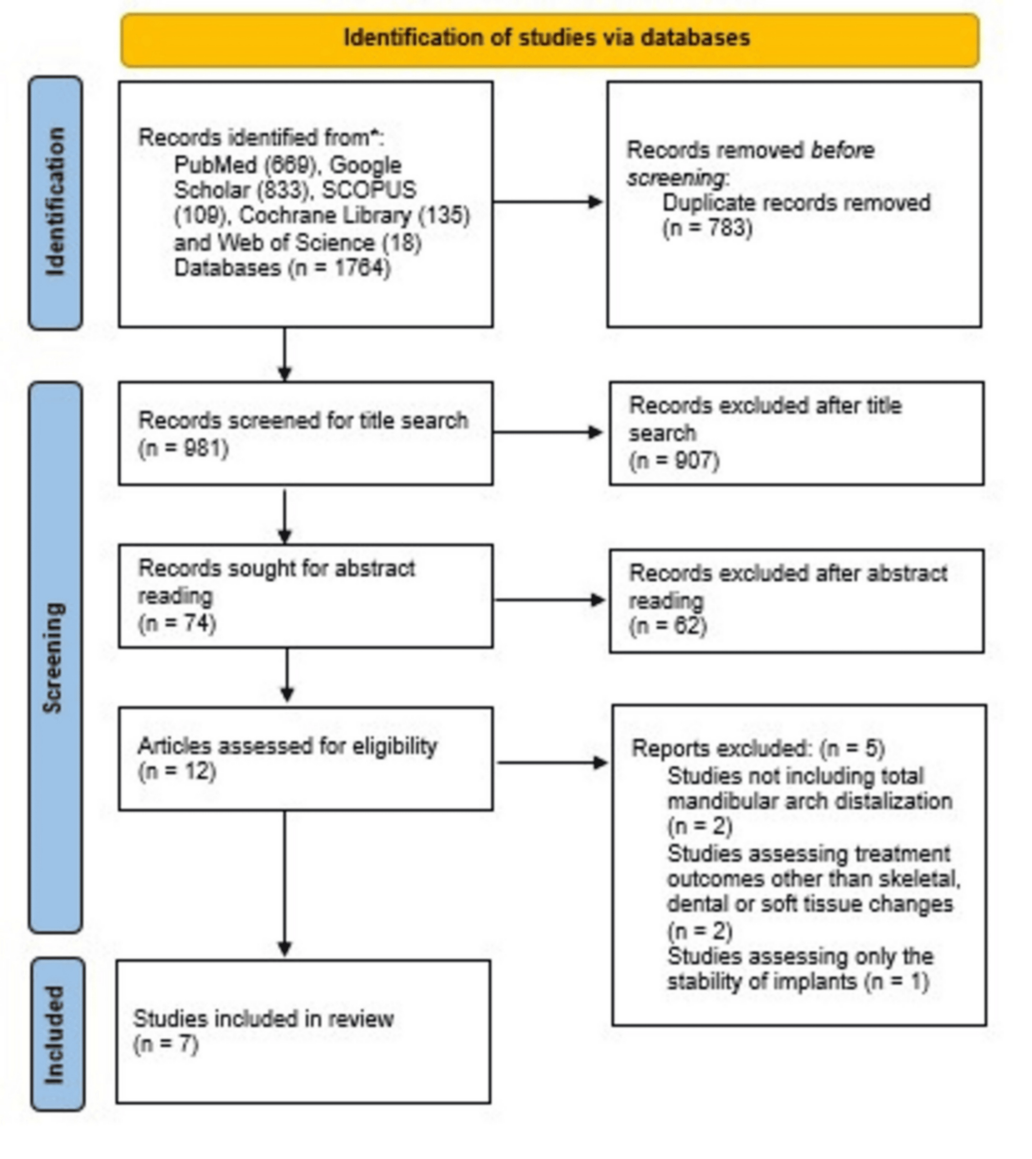
PRISMA flow diagram of included studies. PRISMA: Preferred Reporting Items for Systematic Reviews and Meta-Analyses.

A study was considered eligible when it included distalization of total mandibular arch using a skeletal temporary anchorage system. Studies involving only anterior teeth retraction or distalization by methods other than using TADs were excluded. Duplicates were removed using Rayyan Software (QCRI). Pertinent articles that met the inclusion criteria were filtered based on their title and abstract. To determine which studies would be included in the review, the full texts of the potential articles were accessed and screened when the information was considered insufficient to make a decision. The search for relevant literature, relevance assessment, risk of bias analysis, and extraction of data were carried out. The PRISMA flow diagram illustrates the methodical selection of studies for the review (Figure 1). 

Data extraction

The information obtained from the reviewed research studies is listed in Table 4. 

**Table 4:** Data collection. TADs: temporary anchorage devices.

Data collection
Author
Title
Journal
Year of publication
Study protocol
Number of samples
Sample demographic characteristics
Variety of TADs used
Placement position of TADs
Other methods of molar distalization
Size of mini implants
Investigations used
Appliance and wire used
Force applied
Primary outcome measures
Secondary outcome measures
Statistics used

Disagreements were settled through discussion between the two individuals who independently carried out the data extraction. The data collected included the study’s bibliographic information, information on the study’s design, verification of study eligibility, participant characteristics (number and gender), intervention characteristics (type, location, and size of TADs used, investigations used, appliance and wire used, force applied), and information of outcomes assessed (primary and secondary outcomes, statistics used, and method error assessment). The main results obtained from the articles were the distal and vertical movements of the mandibular molar and incisors, measured in millimeters, along with the angular distal tipping of the same.

Risk of bias/qualitative assessment

The risk of bias in non-randomized studies, which includes observational cohort studies, is assessed using the ROBINS-I tool ("Risk Of Bias In Non-randomized Studies-of Interventions") in the following domains: confounding bias, participant selection, intervention classification, deviations from intended interventions, missing data, outcome measurement, and selection of reported results.

Results

Description of the Included Studies

The PRISMA flow diagram (Figure 1) shows how studies were screened before being included in the review. Following the initial search of five databases, a total of 1764 records were obtained. After the removal of duplicates, title search, and review of abstracts, 12 articles were assessed for eligibility. Five studies were concluded ineligible, of which two studies did not evaluate total mandibular arch distalization; two studies assessed treatment outcomes other than skeletal, dental, or soft tissue changes; and one study assessed only the stability of implants. A total of seven studies were added for qualitative synthesis in this systematic review. The distinct attributes of the included studies are presented in Table 5 and their results in Table 6.

**Table 5:** Individual study characteristics table. M: male; F: female; TADs: temporary anchorage devices; SNB: sella-nasion-B point; ANB: A point-nasion-B point; OP: occlusal plane; MP: mandibular plane; FMA: Frankfort-mandibular plane angle; FH: Frankfort horizontal plane.

Study	Design	Sample size	Intervention	Comparison	Investigations used	Appliance and wires used	Length and diameter of mini implants	Force applied	Parameters assessed and outcomes	Statistics used	Inference
Ye et al., 2013 [16]	Non-RCT	N = 19 (4 M, 15 F)	(n = 10) Miniscrews in retromolar area	(n = 9) Miniscrews in radicular area between maxillary posterior teeth	Lateral cephalographs	0.019x0.025 SS wire on 0.022 in slot pre-adjusted edgewise appliance	d = 1.5 mm, l = 6 mm	200 g per side	Angular measurements(°) ANB SNB Y-axis FMA FH/Occ U1/FH L1/MP L6/MP Linear measurements (mm) U1 to NA L1 to NB Po to NB L1e to MP L1e to RP L1a to RP L6c to MP L6c to RP L6a to RP Overjet Overbite Ls to E-Line Li to E-Line Treatment duration	Paired t-test or Wilcoxon signed rank test for intragroup assessment. Independent t-test or Kolmogorov-Smirnov test for inter-group comparison	Lesser treatment duration and more distal movement seen with retromolar screws compared to maxillary miniscrews. No significant difference in overjet and overbite. Changes in SNB and molar intrusion seen with retromolar screws. Molar extrusion and lingual lower incisor tipping seen with maxillary miniscrews. Favourable soft tissue changes with retromolar screws compared to maxillary screws
Chen et al., 2019 [1]	Retrospective study	N = 20 (10 M, 10 F)	(n = 15) Interradicular miniscrews between lower 5 and 6.	(n = 5) Miniplates in the body of mandible posterior to the dentition	Lateral cephalographs	-	-	-	SN-SS (Mx sagittal) SN-SM (Mn sagittal) S-N-SM (interarch) Overjet Overbite Ils-NL (upper incisor angulation) Ili-ML (lower incisor inclination) Treatment duration	Student's t-test and regression coefficient	No significant difference in the distalization and inclination of teeth between the two groups. Increased extrusion of molar seen with miniplates compared to miniscrews
Yeon et al., 2020 [17]	Retrospective study	N = 40	(n = 20): Interradicular miniscrews between L5 and L6	(n = 20): Ramal plates placed buccal to second molar	Lateral cephalographs	0.019x0.025 SS wire on 0.022 in slot pre-adjusted edgewise appliance	d = 1.5 mm, l = 6 mm	300 g per side	Angular measurements(°) ANB SNB FMA Facial height ratio FH to L occlusal plane Linear measurements (mm) 6C to VFH 6R to VFH 1T to VFH 1R to VFH 6C to MP 6R to MP 1T to MP 1R to MP 6C to VMP 6R to VMP 1T to VMP 1R to VMP 6 to MP 1 to MP Wits appraisal TVL to UL TVL to LL TVL to Pog	Paired t-test and Independent t-test	Greater distalization seen with ramal plates compared to miniscrews at both crown and root level of mandibular molars. Increased retraction and retroclination of the mandibular incisor seen with ramal plates. Buccal miniscrews showed molar intrusion and counterclockwise rotation. Ramal plates showed greater lip retraction and increased FMA
Nakamura et al., 2017 [18]	Retrospective study	N = 23	(n = 11; 2 M, 9 F) Miniscrews, miniplates, and IMF screws on retromolar pads (n=3), between L5 and L6 (n=6), and between L6 and L7 (n=2)	(n = 12; 5 M, 7 F) Class lll elastics without TADs	Lateral cephalographs	0.018x0.025 SS wire in 0.022 in slot pre-adjusted edgewise appliance	-	150-200 g per side	Angular measurements(°) ANB SNA SNB SN-MP y-axis Occ plane U1-SN L1-MP IIA L6-MP Linear measurements (mm) Overjet Overbite L1e to PV L1a to PV L1e to MP L6c to PV L6a to PV L6c to MP U6 to PP Treatment duration	Paired t-test, Student's t-test, and Kruskal-Wallis test	TADs showed distalization and intrusion of first molars. Elastics showed distal tipping and extrusion of first molars. Counterclockwise mandibular rotation seen in TADs group. Clockwise rotation seen in elastic group. No difference in treatment outcomes based on the sites of TAD placement. No significant difference in treatment duration
Song et al., 2022 [11]	Prospective clinical trial	N = 39 (8 M, 31 F)	In mandible: 16 miniscrews and 6 miniplates between L6 and L7, 18 between L5 and L6, and 10 in retromolar area	In maxilla: miniscrews-48 between U5 and U6, 28 between U6 and U7, and two in midpalatal area	Lateral cephalographs	0.016x0.022 SS wire in 0.018 0.025-in slot pre-adjusted edgewise appliances with Roth prescription	-	200 cN	Skeletal SNA(°) SNB(°) ANB(°) SN-OP(°) SN-MP(°) PTV-A(mm) PTV-B(mm) ANS-Me(mm) Facial height ratio(%) Dental linear (mm) U1t-PTV U1r-PTV U6t-PTV U6r-PTV U7t-PTV U7r-PTV U1t-PP U1r-PP U6t-PP U6r-PP U7t-PP U7r-PP Dental angular(°) U1 to SN U6 to SN U7 to SN Soft tissue (mm) Upper lip E-plane Lower lip E-plane	Kolmogorov-Smirnov test of normality, repeated measures ANOVA, and Pearson’s correlation coefficients	Skeletal: Decrease in PTV to Point B. Dental: Significant distalization and distal tipping of lower molars but only retraction of lower incisors and non-significant lingual inclination. No significant change in vertical position. Significant lower lip retraction seen
Oh et al., 2011 [14]	Retrospective study	N = 23	In mandible, miniscrews-14 distobuccal to L7, 26 between L6 and L7, and four between L5 and L6	In maxilla, miniscrews-32 between U5 and U6, six in palatal slope between U6 and U7	Lateral cephalographs	0.017x0.025 SS wire in 0.022-in pre-adjusted edgewise appliance	d = 1.2 mm, l = 6 mm	200 g	Dental changes Angular measurements: MP-L1 MP-L4 MP-L6 MP-L7 Linear measurements: MLC-L4 MLC-L6 MLC–L7 MP-L1 MP-L4 MP-L6 MP-L7	Paired t-test and Wilcoxon signed rank test	Distalized molars show less rotation, intrusion, and distal tipping. Counter clockwise rotation of the mandible seen
Park et al., 2005 [12]	Retrospective study	N = 13	(n = 11) h In mandible, miniscrews-16 distal to L7, four in retromolar area, and two between L6 and L7	(n=4) In maxilla, miniscrews-4 between U5 and U6 and two palatal between U6 and U7	Lateral cephalographs	0.018x0.025 TMA or SS wires in 0.022-in slot pre-adjusted edgewise appliance	d = 1.2, l = 6 mm; d = 1.2, l = 8 mm; d = 2 mm, l = 15 mm	200 g	Skeletal SN to palatal plane SN-OP FH-MP Pterygoid vertical plane to A point Pterygoid vertical plane to B point Lower anterior face height Dental-angular(°) MP-L1 MP-L4 MP-L6 MP-L7 Dental-linear (mm) LC-L4 LC-L6 LC-L7 MP-L1 MP-L4 MP-L6 MP-L7 Soft tissue Ls to E-line Li to E-line	Paired t-test and Wilcoxon signed rank test	Distal tipping of mandibular posteriors with uprighting and retraction of mandibular anteriors seen. Extrusion of first premolars was beneficial in levelling of the curve of Spee

**Table 6:** Results table. TADs: temporary anchorage devices; SNB: sella-nasion-B point; ANB: A point-nasion-B point; OP: occlusal plane; MP: mandibular plane; FMA: Frankfort-mandibular plane angle; FH: Frankfort horizontal plane. Values are presented as mean ± SD.

Parameters	Ye et al., 2013 [16]		Chen et al., 2019 [1]		Yeon et al., 2020 [17]		Nakamura et al., 2017 [18]	Song et al., 2022 [11]	Oh et al., 2011 [14]	Park et al., 2005 [12]
TADs	Retromolar miniscrews	Interradicular miniscrews in the maxilla	Interradicular miniscrews	Miniplates	Interradicular miniscrews	Ramal plates	TADs	TADs	Miniscrews	Miniscrews
Dental										
Molar crown distal movement (mm)	3.6 ± 2.4	3.3 ± 2.6	1.6	3.1	1.9 ± 1.4	2.5 ± 1.4	3 ± 1.4	2.6 ± 4.1	2.5 ± 2.1	2.9 ± 2.1
Molar root distal movement (mm)	1.2 ± 0.7	0.2 ± 0.7			0.4 ± 1.6	1 ± 2.1	0.8 ± 1.4	0.9 ± 4.2		
Molar distal tipping (°)	8.7 ± 5.5	10.7 ± 4.6	6.1	5.4	5.4 ± 4.2	6.8 ± 5.7	6.2 ± 2.9	2.3 ± 6.1	7.6 ± 9.6	5 ± 5.9
Incisor crown distal movement (mm)	2.4 ± 2.6	2.2 ± 2	0.9	0.7	1.7 ± 1.1	3.3 ±1.9	2.6 ± 1.8	0.9 ± 3.4	-	-
Incisor root distal movement (mm)	0.4 ± 1.1	0.1 ± 1.1			0 ± 1.3	0.8 ± 2.1	2.6 ± 1.4	0.5 ± 4.5		
Incisor distal tipping (°)	4.4 ± 2.1	4.9 ± 1.8	1.5	3.1	4.8 ± 5.1	9.2 ± 7.6	0.1 ± 6.5	1.7 ± 5	2.5 ± 5.3	0.8 ± 5.4
Molar vertical movement (mm)	-0.7 ± 0.6	0.2 ± 0.5	0.6	2	-1.3 ± 0.7(crown) -0.7 ± 1.1 (root)	0.2 ± 0.9 (crown) 0.9 ± 1 (root)	-1.2 ± 1.6	-0.3 ± 1.7 (crown) 0.7 ± 2.1 (root)	-0.4 ± 1.1	0.2 ± 1.2
Incisor vertical movement (mm)	0.3 ± 1.1	1.8 ± 1.5	0.6	1.8	0.2 ± 1.6(crown) 0.7 ± 1.8 (root)	0.9 ± 1.4 (crown) 1.5 ± 1.8 (root)	-0.1 ± 2.1	-1.1 ± 2.6 (crown) -0.5 ± 2.4 (root)	0 ± 1.9	0.7 ± 2.1
Overjet (mm)	2.1 ± 1.7		-	-	-	-	3.8 ± 2.5	-	-	-
Overbite (mm)	2.3 ± 1.3		-	-	-	-	0.2 ± 1.7	-	-	-
Skeletal										
SNB (°)	-0.5 ± 0.4	-0.1 ± 0.4	-	-	0.5 ± 1	-0.6 ± 1.3	0 ± 1.3	0.1 ± 0.6	-	-
ANB (°)	-0.6 ± 0.7	-0.1 ± 0.5	-	-	0.4 ± 0.8	0.7 ± 1.1	-0.1 ± 1 1	0.2 ± 0.6	-	-
OP (°)	-4.6 ± 2.3 (with FH)	-4.8 ± 2.9 (with FH)	-	-	-2.2 ± 2.2 (with FH)	0 ± 1.9 (with FH)	-3.2 ± 2.6 (with SN)	0.4 ± 2.6 (with SN)	0 ± 3.3 (with SN)	-1.8 ± 3.3 (with SN)
SN - MP (°)	-	-	-	-	-	-	-0.9 ± 1.6	-0.4 ± 0.9	-	
FMA	-3.3 ± 1.2	1.2 ± 1.1	-	-	-0.2 ± 1.1	0.8 ± 1.1	-	-	-0.7 ± 1.3	0.3 ± 1.5
Mandibular rotation	Counterclockwise	Counterclockwise	-	-	Counterclockwise	Clockwise	Counterclockwise	Counterclockwise	Counterclockwise	Counterclockwise
Soft tissue										
Lower lip retraction (mm)	1.4 ± 0.8	1.2 ± 1	-	-	0.6 ± 1.3	2.3 ± 1.6	-	1.1 ± 1.9	1.2 ± 1.4	1.1 ± 1.8
Treatment duration	-	-	26.5 months	31.8 months	-	-	35.6 ± 12.5 months	24.5 months	20 ± 4.9 months	-
Duration of distalization	6.3 ± 1.5 months	7.6 ± 1.5 months						12.1 months	-	12.3 ± 5.7 months

A total of 127 participants had temporary skeletal anchorage devices placed in the mandible across the seven selected studies. All the included studies had changes in molar distalization and tipping as well as vertical movement of molars and incisors. Five out of the seven studies evaluated incisor distal movement and tipping. Occlusal plane (OP) angle, mandibular plane (MP) angle, and mandibular rotation were measured in six of the seven studies. Lower lip retraction was assessed in five studies. The duration of distalization assessed in four studies.

Risk of Bias Assessment

**Figure 2:**
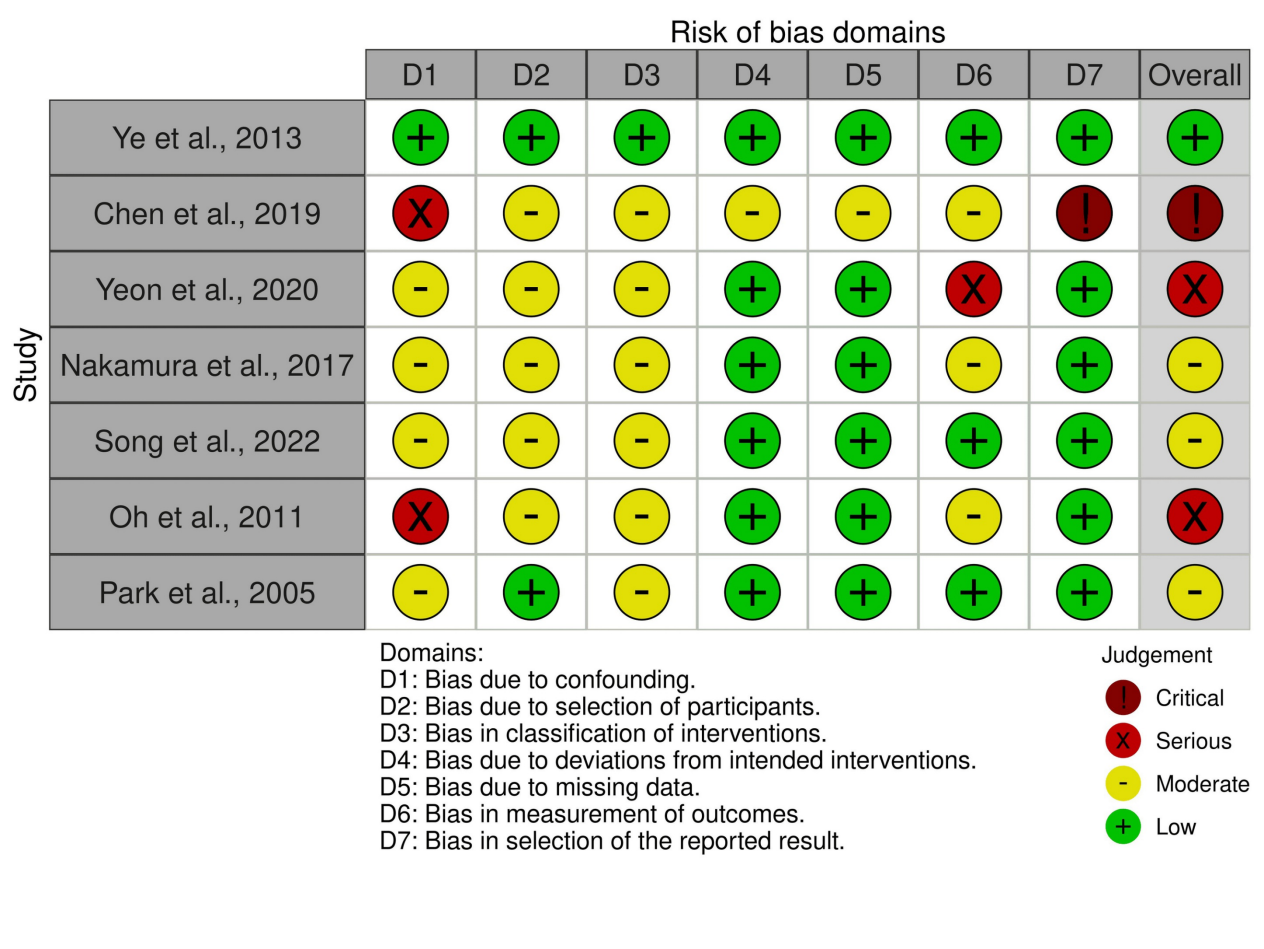
Traffic plot of risk of bias.

**Figure 3:**
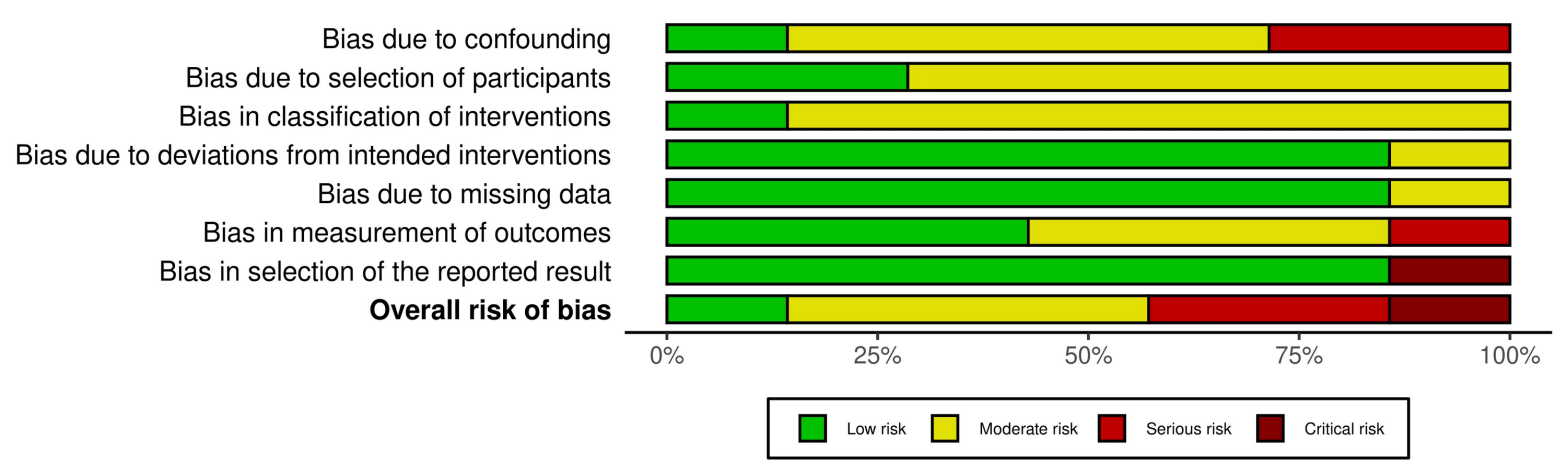
Summary plot of risk of bias.

Of the seven included studies in this review, one was a non-RCT, one was a prospective clinical trial, and five were retrospective cohort studies. All studies were assessed using ROBINS-l tool. One study had a low risk of bias, three had moderate bias, two had serious bias, and one had critical bias (Figures 2, 3) [1,11,12,14,16-18].

Primary Outcomes

Molar distalization: Studies conducted by various authors highlight the varying degrees of molar distalization achieved with different types of miniscrews, demonstrating that the choice of device and its positioning significantly influence the amount of distalization obtained in the crown and root individually. Ye et al. [16] found an average molar distalization of 3.6 ± 2.4 mm (crown) and 1.2 ± 0.7 mm (root) using retromolar miniscrews and 3.3 ± 2.6 mm (crown) and 0.2 ± 0.7 mm (root) using interradicular screws in the maxillary posterior region. These findings were supported by Chen et al. [1] who found 3.1 mm of distalization while using miniplates and 1.6 mm of molar distalization using interradicular miniscrews, and Yeon et al. [17] reported 2.5 ± 1.4 mm (crown) and 1 ± 2.1 mm (root) distalization with ramal plates and 1.9 ± 1.4 mm (crown) and 0.4 ± 1.6 mm (root) distalization with interradicular miniscrews. Similarly, other studies such as Nakamura et al. [18], Song et al. [11], Oh et al. [14], and Park et al. [12] generated comparable results, with miniscrews being the primary mode of distalization utillized. 

Molar distal tipping: The extent of distal tipping of the first molar ranged from 5° to 10°, with multiple studies reporting the same. Ye et al. [16] reported the maximum extent (8.7 ± 5.5° with retromolar screws, 10.7 ± 4.6 with interradicular screws) of tipping. Incidentally, the extent of distalization reported by Ye et al. [16] was also the maximum among the reported studies. In a similar vein, Chen et al. [1] reported 6.1° (miniscrews) and 5.4° (miniplates) and Yeon et al. [17] presented 5.4° (miniscrews) and 6.8° (ramal plates) tipping. Similar findings were reported by Nakamura et al. [18], Song et al. [11], Oh et al. [14], and Park et al. [12].

Molar vertical movement: Molars undergo either intrusion or extrusion during distalization depending on the mechanics used. Yeon et al. [17], Oh et al. [14], and Song et al. [11] showed molar intrusion with interradicular miniscrews. Molar intrusion was also observed with retromolar miniscrews by Ye et al. [16]. Conversely, Chen et al. [1] observed molar extrusion when using interradicular screws and miniplates, which was consistent with results from Yeon et al. [17] (ramal plates), Park et al. [12] (miniscrews), and Ye et al. [16] (maxillary miniscrews).

Incisor distalization: Incisor distalization is another key outcome influenced by the type of TAD used. Ye et al. [16] reported distal movement of 2.4 ± 2.6 mm (crown) and 0.4 ± 1.1 mm (root) with retromolar screws and 2.2 ± 2 mm (crown) and 0.1 ± 1.1 mm (root) with maxillary interradicular screws. Chen et al. [1] reported lesser incisor distalization of 0.9 mm by interradicular miniscrews and 0.7 mm by miniplates compared to Yeon et al. [17] who showed 1.7 ± 1.1 mm (crown) and 0 ± 1.3 mm (root) distalization with interradicular miniscrews and 3.3 ± 1.9 mm (crown) and 0.8 ±2.1 mm (root) distalization with ramal plates. Studies by Nakamura et al. [18] and Song et al. [11] provided varying results, with Nakamura et al. [18] showing the highest distalization (2.6 ± 1.8 mm crown, 2.6 ± 1.4 mm root) while Song et al. [11] evidenced the least distalization values (0.9 ± 3.4 mm crown, 0.5 ± 4.5 mm root).

Incisor distal tipping: All the studies showed varying levels of incisal tipping ranging from 0.2 ± 1.2° with miniscrews by Park et al. [12] to 9.2 ± 7.6° with ramal plates by Yeon et al. [17]. Interradicular miniscrews produced similar tipping in studies by Ye et al. [16] (4.9 ± 1.8°) and Yeon et al. [17] (4.8 ± 5.1°). Other TADs, such as retromolar miniscrews, miniplates, and ramal plates, exhibited a varied range of tipping.

Incisor vertical movement: All the included studies reported incisor extrusion during distalization, with only Nakamura et al. [18] and Song et al. [11] observing incisor intrusion.

Secondary Outcomes

Dental changes (overjet and overbite): Ye et al. [16] reported a difference of 2.1 ± 1.7 mm in overjet and 2.3 ± 1.3 mm in overbite. Nakamura et al. [18] reported a change of 3.8 ± 2.5 mm in overjet and 0.2 ± 1.7 mm in overbite.

Skeletal changes (SNB and ANB): Alterations in the SNB and ANB angles were noted in all studies. A decrease in both SNB and ANB was reported by Ye et al. [16], while an increase in these angles was observed with interradicular screws by Yeon et al. [17] and Song et al. [11]. Yeon et al. [17] also reported a decrease in SNB but an increase in ANB with ramal plates. Nakamura et al. [18] noted an increase in SNB but a decrease in ANB with TADs.

OP angle: An anticlockwise rotation of the OP was seen with interradicular screws in studies by Ye et al. [16], Yeon et al. [17], Nakamura et al. [18], and Park et al. [12]. Conversely, a clockwise rotation of the OP was seen with ramal plates in studies by Yeon et al. [17], Song et al. [11], and Oh et al. [14].

MP angle: A decrease in the MP angle was seen with interradicular screws in studies by Ye et al. [16], Yeon et al. [17], and Oh et al. [14]. An increase in the MP angle was seen with miniplates by Ye et al. [16], ramal plates by Yeon et al. [17], and miniscrews by Park et al. [12].

Mandibular rotation: The mandible rotates anticlockwise, as observed in all studies except in the one by Yeon et al. [17] using ramal plates.

Soft tissue (lower lip retraction): All studies consistently reported significant retraction of the lower lip as a secondary soft tissue effect of distalization, regardless of the type of anchorage used.

*Treatment duration:* A shorter duration of distalization is seen with retromolar screws and ramal plates compared to interradicular screws, likely due to the greater efficiency in distalizing the molars with these devices.

Discussion

The use of skeletal TADs for en masse mandibular arch distalization has proven to be a viable option for treating borderline Class III malocclusion cases without the need for surgical intervention [8]. The systematic review analysed here provides a comprehensive examination of the existing literature, identifying significant treatment effects and differences between various TAD types.

The studies included in the review consistently demonstrated that TADs are effective in achieving mandibular arch distalization, which is crucial for the correction of Class III malocclusions. This supports previous findings that TADs provide stable anchorage, leading to predictable and controlled tooth movements without relying on patient compliance [19-22].

Magnitude of Distalization

The extent of molar distalization varied between studies, with some reporting higher average distalization than others. This discrepancy could be due to differences in study design, sample size, TAD placement sites, and patient characteristics [16,18].

Distalization and Distal Tipping

A critical aspect of evaluating TAD efficacy is understanding the type of tooth movement achieved. Distalization of the mandibular dentition involves differentiating between bodily movement and tipping. Bodily movement, characterized by more root movement and less tipping, is preferable for achieving stable outcomes [23]. Changes in distal movement, vertical movement, or tipping must be specified.

From the studies included in this review, more distal tipping is seen with retromolar screws compared to ramal plates. The least tipping is seen with interradicular screws placed between the lower second premolar and first molar. Great distalization of the molars with higher molar root movement is observed with ramal plates compared with retromolar screws and interradicular screws. Therefore, ramal plates are more efficient in molar distalization with less tipping and more bodily movement. These findings are in accordance with previous studies by Kook et al., Yu et al., and Yeon et al. [6,7,17]. The ramal plate's design allows for better control of the force vectors, leading to more favorable outcomes in terms of root movement and overall distalization. 

The amount of incisor distalization is lower compared to molar distalization. Greater distal tipping of the incisors is observed in the included studies. The distal movement is due to the distal tipping of the incisors rather than the bodily movement of the teeth, as reduced distal movement is seen at the incisor apex level. Anterior teeth round tripping is greatly reduced with TADs with minimal loss of anterior anchorage [18,24].

Vertical Movements

Depending on the type of anchorage devices used, the incisors and molars were positioned substantially different in the vertical dimension. Buccal miniscrews showed intrusion of the molars, while ramal plates and maxillary screws showed more extrusion of the lower molars. Therefore, miniscrews produced an anticlockwise rotation of the mandible, whereas miniplates produced a clockwise rotation. The wedging effect of molar distalization is compensated by intrusion. Incisor extrusion was observed in all studies except those by Nakamura et al. and Song et al. Desired outcomes can be achieved by choosing the TAD variety based on the growth pattern of Class III patients [25]. When vertical force vectors are applied correctly, teeth can distalize more effectively and lower molar tipping can be avoided [26].

Overjet, Overbite, and Facial esthetics

There is a significant change in the overjet and overbite of the patients after distalization. For Class lll patients, mandibular backward rotation may be beneficial in reducing overjet [16]. The levelling of the curve of Spee and uprighting of the OP occur by intrusion and distal tipping of the lower molars and extrusion of the premolars [12,14]. Significant retraction of the lower lips was seen in all studies after anterior teeth retraction. Hence, TADs help achieve a balanced facial esthetics in borderline cases without the need for surgery [27].

Efficiency and Treatment Duration

The treatment effects of TADs for the en masse distalization of lower teeth were measured in this study. There is significant distal movement of the lower molars, more with miniplates when compared to miniscrews. Lower duration is required for distalization with retromolar screws and ramal plates compared to interradicular screws. With TADs, it is possible for en masse distalization of all teeth together with decreased distal tipping and rotation of molars, leading to upper and lower lip retraction resulting in an improved facial profile [28]. Therefore, TADS are efficient in treating borderline patients without extractions [29].

Limitations

The included studies lacked quantification of the distalization that occurred in specified periods of time. Cases of unilateral distalization were not mentioned and the influence of simultaneous distalization of the maxilla on the mandible were not taken into account. In-depth information on the vectors of force of the TADs at specified locations was unavailable. The failure rates, along with primary and secondary stability of the implants, are confounding factors affecting the treatment outcomes and should be taken into consideration. A meta-analysis could not be performed because the area of interest is relatively new, with limited data and heterogeneity among the evaluated parameters. 

## Conclusions

In conclusion, the key findings in this systematic review are that miniplates and retromolar screws placed distal to the mandibular dentition are more effective in distalization compared to interradicular screws. However, greater distal tipping occurs with retromolar screws compared to ramal plates, with the least tipping seen in interradicular screws. Intrusion of molars occurred with interradicular screws, whereas extrusion occurred with ramal plates. Therefore, the selection of TADs should be based on the patients' growth pattern. The expected wedging effect due to distalization of the dentition is compensated by molar intrusion, leading to mandibular rotation in an anticlockwise direction. Significant retraction of the lower lips was observed in all studies. Thus, TADs help achieve a harmonious facial profile in borderline cases without the need for surgery.

## References

[1] Chen J, Patino JI, Chang JZ, Yao CC, Nielsen IL (2019). A comparison of two TAD techniques (miniscrews versus miniplates) for treating Class III malocclusion and the associated skeletal and dental effects. Taiwan J Orthod.

[2] Kim YH, Han UK, Lim DD, Serraon ML (2000). Stability of anterior openbite correction with multiloop edgewise archwire therapy: a cephalometric follow-up study. Am J Orthod Dentofacial Orthop.

[3] Saito I, Yamaki M, Hanada K (2005). Nonsurgical treatment of adult open bite using edgewise appliance combined with high-pull headgear and class III elastics. Angle Orthod.

[4] Liu H, Li JX (2013). Non-surgical treatment of an Angle Class III malocclusion in adults. Int J Clin Exp Med.

[5] Kuroda Y, Kuroda S, Alexander RG, Tanaka E (2010). Adult Class III treatment using a J-hook headgear to the mandibular arch. Angle Orthod.

[6] Kook YA, Park JH, Bayome M, Kim S, Han E, Kim CH (2016). Distalization of the mandibular dentition with a ramal plate for skeletal Class III malocclusion correction. Am J Orthod Dentofacial Orthop.

[7] Yu J, Park JH, Bayome M, Kim S, Kook YA, Kim Y, Kim CH (2016). Treatment effects of mandibular total arch distalization using a ramal plate. Korean J Orthod.

[8] Poletti L, Silvera AA, Ghislanzoni LTH (2013). Dentoalveolar class III treatment using retromolar miniscrew anchorage. Prog Orthod.

[9] Ohura R, Kuroda S, Takahashi T, Tomita Y, Tanaka E (2011). Efficient usage of implant anchorage to treat overerupted maxillary first molar and mesially inclined mandibular molars. Am J Orthod Dentofacial Orthop.

[10] Chung K, Kim SH, Kook Y (2005). C-orthodontic microimplant for distalization of mandibular dentition in Class III correction. Angle Orthod.

[11] Song BJ, Lee KJ, Cha JY, Lee JS, Mo SS, Yu HS (2022). Stability of the maxillary and mandibular total arch distalization using temporary anchorage devices (TADs) in adults. Appl Sci.

[12] Park HS, Lee SK, Kwon OW (2005). Group distal movement of teeth using microscrew implant anchorage. Angle Orthod.

[13] Sugawara J, Daimaruya T, Umemori M, Nagasaka H, Takahashi I, Kawamura H, Mitani H (2004). Distal movement of mandibular molars in adult patients with the skeletal anchorage system. Am J Orthod Dentofacial Orthop.

[14] Oh YH, Park HS, Kwon TG (2011). Treatment effects of microimplant-aided sliding mechanics on distal retraction of posterior teeth. Am J Orthod Dentofacial Orthop.

[15] Chung KR, Kim SH, Choo H, Kook YA, Cope JB (2010). Distalization of the mandibular dentition with mini-implants to correct a Class III malocclusion with a midline deviation. Am J Orthod Dentofacial Orthop.

[16] Ye C, Zhihe Z, Zhao Q, Ye J (2013). Treatment effects of distal movement of lower arch with miniscrews in the retromolar area compared with miniscrews in the posterior area of the maxillary. J Craniofac Surg.

[17] Yeon BM, Lee NK, Park JH, Kim JM, Kim SH, Kook YA (2022). Comparison of treatment effects after total mandibular arch distalization with miniscrews vs ramal plates in patients with Class III malocclusion. Am J Orthod Dentofacial Orthop.

[18] Nakamura M, Kawanabe N, Kataoka T, Murakami T, Yamashiro T, Kamioka H (2017). Comparative evaluation of treatment outcomes between temporary anchorage devices and Class III elastics in Class III malocclusions. Am J Orthod Dentofacial Orthop.

[19] Sreenivasagan S, Subramanian AK, Rengalakshmi S (2021). Prevalence and cause of mini-implant failure encountered by orthodontic residents. J Long Term Eff Med Implants.

[20] Schätzle M, Männchen R, Zwahlen M, Lang NP (2009). Survival and failure rates of orthodontic temporary anchorage devices: a systematic review. Clin Oral Implants Res.

[21] Park JH (2020). Temporary Anchorage Devices in Clinical Orthodontics. John Wiley & Sons.

[22] Chang C, Liu SS, Roberts WE (2015). Primary failure rate for 1680 extra-alveolar mandibular buccal shelf mini-screws placed in movable mucosa or attached gingiva. Angle Orthod.

[23] Smith RJ, Burstone CJ (19841). Mechanics of tooth movement. Am J Orthod.

[24] Arvind PT, Ramasamy N, Rengalakshmi S (2021). Comparative evaluation of anchorage loss with implant-aided retraction and frictionless mechanics with conventional anchorage in bimaxillary protrusion cases. J Long Term Eff Med Implants.

[25] Venugopal A, Manzano P, Vaid NR (2022). TAD driven Class III camouflage: eight point protocol to optimize efficiency, aesthetics and stability. Semin Orthod.

[26] Sung EH, Kim SJ, Chun YS, Park YC, Yu HS, Lee KJ (2015). Distalization pattern of whole maxillary dentition according to force application points. Korean J Orthod.

[27] Mahmood TM (2023). Occlusal plane steepness and profile change following TAD-based one-step retraction on four-unit extraction cases: a retrospective study. Diagnostics (Basel).

[28] Shaikh A, Jamdar AF, Galgali SA, Patil S, Patel I, Hemagiriyappa MS (2021). Efficacy of infrazygomatic crest implants for full-arch distalization of maxilla and reduction of gummy smile in class II malocclusion. J Contemp Dent Pract.

[29] Olivieri P, Uribe FA, Quereshy FA (2020). Aesthetic facial surgery and orthodontics: common goals. Oral Maxillofac Surg Clin North Am.

